# Potential biomarkers and immune infiltration linking endometriosis with recurrent pregnancy loss based on bioinformatics and machine learning

**DOI:** 10.3389/fmolb.2025.1529507

**Published:** 2025-02-03

**Authors:** Jianhui Chen, Qun Li, Xiaofang Liu, Fang Lin, Yaling Jing, Jiayan Yang, Lianfang Zhao

**Affiliations:** ^1^ Prenatal Diagnosis Center, Center of Reproductive Medicine, Suining Central Hospital, Suining, Sichuan, China; ^2^ Department of Radiology, Suining Central Hospital, Suining, Sichuan, China

**Keywords:** endometriosis, immune infiltration, recurrent pregnancy loss, biomarker, endometrial cancer

## Abstract

**Objective:**

Endometriosis (EMs) is a chronic inflammatory disease characterized by the presence of endometrial tissue in the non-uterine cavity, resulting in dysmenorrhea, pelvic pain, and infertility. Epidemiologic data have suggested the correlation between EMs and recurrent pregnancy loss (RPL), but the pathological mechanism is unclear. This study aims to investigate the potential biomarkers and immune infiltration in EMs and RPL, providing a basis for early detection and treatment of the two diseases.

**Methods:**

Two RPL and six EMs transcriptomic datasets from the Gene Expression Omnibus (GEO) database were used for differential analysis via limma package, followed by weighted gene co-expression network analysis (WGCNA) for key modules screening. Protein-protein interaction (PPI) network and two machine learning algorithms were applied to identify the common core genes in both diseases. The diagnostic capabilities of the core genes were assessed by receiver operating characteristic (ROC) curves. Moreover, immune cell infiltration was estimated using CIBERSORTx, and the Cancer Genome Atlas (TCGA) database was employed to elucidate the role of key genes in endometrial carcinoma (EC).

**Results:**

26 common differentially expressed genes (DEGs) were screened in both diseases, three of which were identified as common core genes (MAN2A1, PAPSS1, RIBC2) through the combination of WGCNA, PPI network, and machine learning-based feature selection. The area under the curve (AUC) values generated by the ROC indicates excellent diagnostic powers in both EMs and RPL. The key genes were found to be significantly associated with the infiltration of several immune cells. Interestingly, MAN2A1 and RIBC2 may play a predominant role in the development and prognostic stratification of EC.

**Conclusion:**

We identified three key genes linking EMs and RPL, emphasizing the heterogeneity of immune infiltration in the occurrence of both diseases. These findings may provide new mechanistic insights or therapeutic targets for further research of EMs and RPL.

## 1 Introduction

Endometriosis (EMs) is an estrogen-dependent chronic gynecological inflammatory disease. Its most typical clinical symptoms are endometrial tissues in non -uterine cavity (Mikhaleva et al., 2020). About 10% of childcare women suffer from EMs. The reason for EMs currently accepted is that the endometrium tissue is retrograde in the pelvis with menstruation, scattered to different areas, producing local inflammatory reactions, causing local tissue adhesion, and then dysmenorrhea, and dysmenorrhea. EMs can lead to multiple clinical symptoms such as pelvic pain and infertility (Barnhart et al., 2002). The prevalence of EMs among women of reproductive age is largely due to the lack of effective screening methods in the early days. EMs often has two treatments in clinical practice: first, drug treatment, oral hormone drug treatment, this treatment can only help EMs patients to relieve symptoms. When drug treatment is interrupted, the probability of recurrence. Second, surgical treatment, an endometrium tissue with abnormal hyperplasia through laparoscopic surgery, separated the adhesive part, but surgery may cause the ovarian reserve function to decrease, and the risk of recurrence is higher. Although EMs is a benign gynecological disease, the pain caused to patients and the risk of a certain vicious transformation of patients brings a lot of trouble to many patients with childbearing age (Barreta et al., 2018). At present, in clinical practice, an efficient diagnosis and treatment plan is needed.

The stability of the function and environment of the uterus and its accessories is related to the stability of pregnancy and the quality of pregnancy. Clinically, embryo or fetal loss of twice or more than two or more pregnancy failure of pregnancy before 20–24 weeks of pregnancy is diagnosed with recurrent pregnancy loss (RPL, Recurrent Pregnancy Loss), and 5% of women try to experience this pain when they are pregnant (Bohlmann et al., 2010; Practice Committee of the American Society for Reproductive Medicine, 2020). The literature reports about 23 million abortions each year, and the prevalence of recurrent abortion is about 2.6% (Quenby et al., 2021). RPL not only brings tremendous psychological pressure to both husband and wife, but it also brings a heavy burden on family, social, and public health systems. The cause of RPL is complicated, and about 50% of the cause of RPL is unknown (Li et al., 2023). The onset of RPL includes many factors such as uterus and accessories, endocrine, immune, genetic, environmental, lifestyle, psychological conditions, etc., (Dimitriadis et al., 2020; Liu et al., 2021). The normal and stability of the environment in the uterus and the accessories are crucial. Due to the lack of a clear understanding of the RPL mechanism, the treatment of patients with RPL is more complicated. A survey of epidemiological diseases found that nearly half of the patients were related to EMs, and many patients caused the failure of auxiliary reproduction (Homer, 2019).

To explore the risk factors of RPL and tap their pathogenesis, more and more researchers have explored the relationship between genetic factors and RPL. However, there is no high-quality monitoring and treatment method for RPL. We focus on EMs, using multiple bioinformatics and machine learning approaches, such as WGCNA, PPI network construction, function enrichment, immune infiltration deconvolution, etc., To reveal the common key biomarkers and their functions in EMs and RPL. A total of eight microarray datasets including 291 samples were enrolled from the gene expression Omnibus (GEO) database. The potential upstream regulatory factors of the biomarkers, i.e., miRNAs or transcription factors were predicted. Finally, we unveiled the pivotal role of two key genes during the development and prognosis of endometrial carcinoma. All these data offer an insightful understanding of molecular mechanisms in EMs and RPL, providing promising directions for early detection of EM, and future extensive studies of these biomarkers may shed light on drug candidate discovery and optimized clinical plans for system risk assessment and personalized therapy of RPL.

## 2 Materials and methods

### 2.1 Data source

We retrieved eight datasets from the GEO database, including two RPL datasets (accessing ID: GSE26787 and GSE165004) and six EMs datasets (accessing ID: GSE51981, GSE6364, GSE7305, GSE120103, GSE23339, and GSE7846). Detailed information on these datasets can be found in Supplementary Table S1. All microarray data preprocessing was performed as previously described (Zhang et al., 2021; Tang et al., 2022b). The “Combat” function implemented in the “SVA” package (v3.42.0), which is a popular algorithm for adjusting systematic variations between arrays, was utilized to generate a combined meta-dataset by removing the batch effects of different sets for both RPL and EMs (Zhang et al., 2023; Guo et al., 2024), respectively, which was then used to perform subsequent bioinformatics analysis. The online TIMER2.0 database (http://timer.cistrome.org/) was used to compare the expression levels of the key genes. Additionally, the RNA-seq profile and corresponding clinical information of EC patients (TCGA-UCEC) were acquired from the UCSC XENA database (https://xena.ucsc.edu) (Guo et al., 2021; Zhao et al., 2023), and log2 transformed Transcripts per kilobase million (TPM) value was computed for normalization (Guo et al., 2022; Tang et al., 2022b).

### 2.2 DEGs screening

DEGs in EMs and RPL were screened using the R package “Limma” (v3.50.3) with the threshold of |logFC| >0.585 and adj.P.Val <0.05 (Sun W. et al., 2023). Considering the imbalanced sample composition for EMs, we utilized the subset-based approach to create the 1:1 ratio for the disease and control groups (Tang et al., 2022a). The common DEGs derived from all five subsets were intersected by drawing a Venn diagram, which were defined as the DEGs of EMs. Subsequently, we obtained the intersection of DEGs from EMs and RPL and visualized their expression patterns using heatmap via the “pheatmap” package (v1.0.12).

### 2.3 WGCNA analysis

Based on the combined datasets of RPL and EMs, the “WGCNA” package (v1.71) was applied to define functional transcriptomic co-expression modules shared by both diseases. Outlier samples were excluded according to the sample clustering plot, followed by the selection of the best soft thresholding power (β) and the establishment of a scale-free network. Dynamic and merged gene modules were generated according to the module size and similarity. The module-trait relationships were determined by Pearson correlation analysis to identify important gene modules, and gene significance (GS) and module membership (MM) were evaluated and scatter plots were depicted.

### 2.4 PPI network construction and function enrichment

PPI analysis was conducted by the STRING database (version 12.0, http://string-db.org/) with the confidence score of ≥0.25 and was further visualized by the Cytoscape software (v3.2.1). The MCODE app was used for cluster analysis. The topological parameters of the network including node degree and maximal clique centrality (MCC) were computed by the “cytohubba” application, which were used to pick out the core genes of the network.

For a better understanding of the potential biological function of identified gene modules from WGCNA, GO and KEGG pathway enrichment analyses were performed using the Metascape (http://metascape.org) with default settings.

### 2.5 Machine learning algorithms to discover potential diagnostic markers

Two machine learning algorithms including the least absolute shrinkage and selection operator (LASSO) regression and support vector machine-based recursive feature elimination (SVM-RFE) were adopted for feature selection to identify candidate diagnostic biomarkers in both RPL and EMs. The expression of the common DEGs and core genes of the PPI network in the combined dataset served as the input matrices for both RPL and EMs. LASSO was carried out by the “glmnet” package (v4.1.8) and SVM-RFE was carried out by the “e1071” package (v1.7.16). Ten-fold cross-validation was applied to calculate the misclassification error of the candidate model and to determine the optimal lambda. λ.1se was used to pick out the least number of variables for LASSO. For SVM-RFE, a ranked gene list was created using the SVM-RFE algorithm, followed by the optimal subset acquisition with a linear kernel. We obtained the intersection of LASSO and SVM-RFE to define the core genes for RPL and EMs, respectively, and the overlapping core genes of the two diseases were further regarded as common core genes representing diagnostic biomarkers of patients with EMs and RPL.

### 2.6 Diagnostic/therapeutic value assessment and immune infiltration analysis

Based on the combined datasets, the “pROC” package (v1.18.4) was used to depict the ROC curves to assess the diagnostic values of the common core genes in patients with EMs and RPL. Besides, Drug SlGnatures DataBase (DSigDB, v1.0), which is a comprehensive gene set resource consisting of 17,389 unique compounds covering 19,531 genes, was used to predict potential drugs/compounds that may disturb the expression of the identified core biomarkers in both EMs and RPL. For immune infiltration estimation, we utilized the CIBERSORTx algorithm (Newman et al., 2019) to obtain the abundance of immune cells in RPL and EMs based on the combined datasets of the two diseases. The immune infiltration landscape in different groups of RPL or EMs was presented by a heatmap plot. Spearman correlation analysis was used to assess the relationship between the expression of the common core genes and immune cell infiltration.

### 2.7 Upstream regulation prediction

MiRNet 2.0 is an up-to-date integrated platform that illustrates “multiple-to-multiple” relationships and functional interpretation. In this study, MiRNet 2.0 was applied to examine the upstream regulation of the abovementioned common core genes. Predicted transcription factor-key gene interactions were inferred from the Jaspar database that plugged in MiRNet 2.0. Moreover, miRNAs that may target these common core genes were predicted with miRTarBase 9.0, an online database to predict miRNA-target interactions (MTIs) which were verified by series types of cell-based experiments including microarray, CLASH, PAR-CLIP, HITS-CLIP, and proteomics. The interactions between predicted transcription factors or miRNAs with common core genes were displayed by Cytoscape.

### 2.8 Comprehensive exploration of the common core genes in EC

TIMER 2.0 was used to compare the expression levels of the common core genes between tumor and adjacent normal tissues in pan-cancer based on the whole TCGA cohort. The TCGA-UCEC dataset was used to assess the correlation between gene expression and clinicopathological features including age, BMI, grade, histology, stage, and tumor burden. Immune score and stromal score were computed with the ESTIMATE algorithm (Yoshihara et al., 2013) via the “estimate” package (v1.0.13). Kaplane-Meier survival plots for overall survival (OS) and disease-free survival (DFS) were drawn to evaluate the prognostic power of common core genes in EC.

### 2.9 Human samples collection

Three samples of ectopic endometrial lesions were collected from women aged 18–37 years, and the diagnosis was confirmed by an experienced histopathologist. Three proliferative-phase endometrial specimens were collected from regular cycling women undergoing hysterectomy for non-endometrial conditions. The tissues were fixed in formalin and embedded in paraffin for immunohistochemistry. The written informed consent for use of human tissues was obtained from each subject, and ethical approval was obtained from the Suining Centre Hospital (No. KYLLKS20240183).

### 2.10 Immunohistochemistry

Tissue sections (5 μm) were dewaxed, EDTA antigen repair solution (MXB, MVS-0099) for antigen thermal repair, 3% H2O2 quenches endogenous peroxidase, 1% BSA blocked 20 min at room temperature, washed with ddH2O and PBS, and then probed with the primary antibody (RIBC2, Proteintech, Rosemont, IL 60018, United States; MAN2A1, HUABIO Hangzhou, Zhejiang P.R.C; PAPSS1, Proteintech, Rosemont, IL 60018, United States) at 1/100 dilution overnight at 4°C. Following washing with 0.025% Tween/Tris, sections were incubated with horseradish peroxidase (HRP)–labeled streptavidin-biotin detection kits according to the manufacturer’s instructions (Dako, Carpinteria, CA). The color was developed by incubation with DAB chromagen (3, 3′- diaminobenzidine; Dako, Carpinteria, CA) for 5 min.

## 3 Results

**FIGURE 1:**
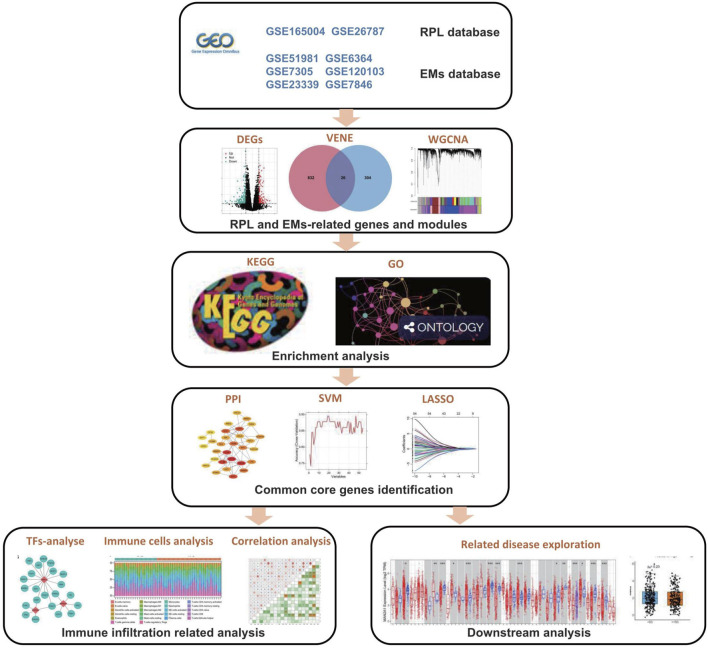
Flowchart of the current research.

We downloaded two RPL datasets and six EMs datasets from the GEO database, respectively. After batch removal in individual diseases, we obtained two combined datasets for RPL and EMs, which were further used for downstream analysis. The principal component analysis (PCA) plots indicated that batch effects were well corrected for both EMs and RPL (Supplementary Figure S1). The combined RPL set was composed of 29 RPL and 29 control tissue samples, while the combined EMs set consisted of 141 EMs and 92 control tissue samples. The complete flowchart of the current research is presented in Figure 1.

### 3.1 DEGs screening in RPL and EMs

**FIGURE 2:**
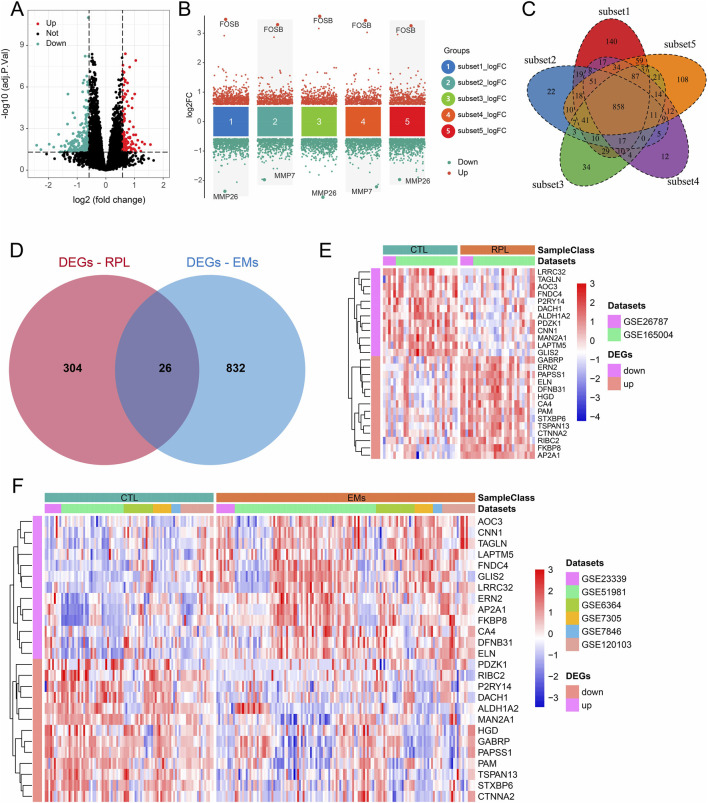
DEGs screening in RPL and EMs. **(A)** Volcano plots illustrating DEGs between RPL and control in the combined RPL dataset. **(B)** Screening of the DEGs between EMs and control by the subset-based approach. **(C)** Venn diagram showing the 858 DEGs shared by the subset-based DEGs. **(D)** Venn diagram showing the 26 common DEGs of RPL and EMs. **(E, F)** The expression of all 26 DEGs in the combined RPL datast **(E)** and EMs dataset **(F)**.

Using the combined datasets of RPL and EMs, we screened the DEGs in these two diseases with the predefined filtering criteria (abs (log2FC) > 0.585, adj.P.Val<0.05). For RPL, a total of 330 DEGs were achieved between 29 pairs of RPL and control samples (Figure 2A). For EMs, we first screened the DEGs between five subsets EMs (n = 92) and control (n = 92). Then, we intersected the DEGs to get 858 DEGs (Figures 2B, C). Next, overlapping analysis of the Venn diagram resulted in 26 common DEGs of RPL and EMs (Figure 2D). The expression patterns of these common DEGs in two combined datasets of RPL and EMs were shown by heatmap (Figures 2E, F).

### 3.2 WGCNA to identify key modules in RPL and EMs

**FIGURE 3:**
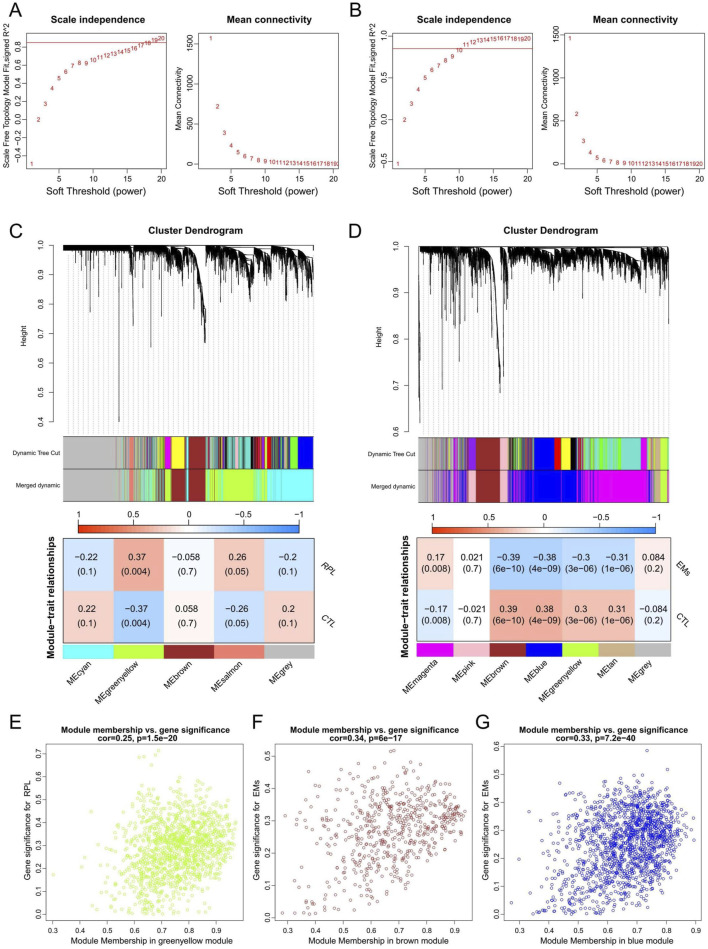
WGCNA to identify key gene modules in RPL and EMs. **(A, B)** The selection of the optimal soft-threshold power in RPL **(A)** and EMs **(B)**. **(C, D)** Gene cluster dendrograms and correlation analysis of module eigengenes of RPL **(C)** and EMs **(D)**. **(E)** Scatter plot illustrating the relationship between GS and MM in the greenyellow module of the combined RPL dataset. **(F, G)** Scatter plots of the brown **(F)** or blue **(G)** gene modules illustrating the relationship between GS and MM in the combined EMs dataset, respectively.

WGCNA network was constructed to unveil the co-expression gene patterns and identify key modules related to EMs or RPL. Outlier samples were screened by sample clustering for both EMs and RPL (Supplementary Figure S2), followed by the determination of optimal β values for meaningful scale-free topology (Figures 3A, B). Subsequently, genes in the RPL and EMs dataset were clustered into 5 and 7 modules, respectively. Pearson correlation analysis revealed that the greenyellow gene module was mostly positively related to RPL (r = 0.37, p = 0.004), while the brown and blue gene modules were mostly negatively connected with EMs (r = −0.39, p = 6e-10; r = −0.38, p = 4e-10) (Figures 3C, D). Besides, scatter plots of these modules were presented with GS and MM in Figures 3E–G.

### 3.3 Function enrichment and pathway analysis of the three key gene modules in RPL and EMs

**FIGURE 4:**
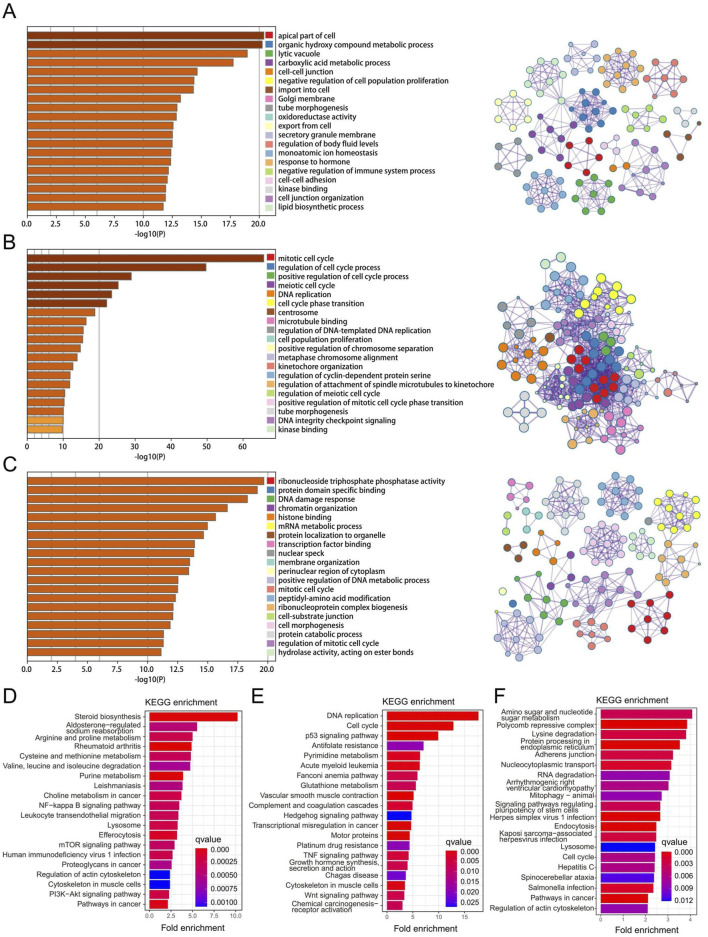
Function enrichment and pathway analysis of the three key gene modules in RPL and EMs. **(A–C)** GO enrichment analysis of the greenyellow module in RPL **(A)**, the blue **(B)** and the brown **(C)** modules in EMs. **(D–F)** KEGG pathway enrichment analysis of the greenyellow module in RPL **(D)**, the blue **(E)** and the brown **(F)** modules in EMs.

We obtained three key gene modules in RPL and EMs via WGCNA, including the greenyellow gene module in RPL and the brown and blue modules of EMs. To understand the biological functions of three key gene modules in RPL and EMs, we performed GO and KEGG analysis. We displayed the top 20 results of GO biological process enrichment analysis with the green-yellow gene module related to RPL and the brown and blue gene modules related to EMs, respectively (Figures 4A–C). To further investigate the link between these GO enrichment results of RPL and EMs, we took the intersection. A total of 67 were retrieved, focused on three GTPase-related results, including GTPase regulator activity, GTPase activator activity, GTPase activity and protein tyrosine kinase activity, ribonucleoside triphosphate phosphatase activity, glycosyltransferase activity, scaffold protein binding, and transcription factor binding, etc. On the other hand, KEGG pathway enrichment analysis found that the greenyellow gene module was mostly involved in 96 pathways, while the blue gene module was major related to 76 pathways. Moreover, the brown gene module was closely related to 69 pathways. The top 20 enriched KEGG pathways of three key gene modules in RPL and EMs are shown in Figures 4D–F. The intersection of KEGG results of three key gene modules indicated that the PI3K-Akt signaling pathway, pathways in cancer, p53 signaling pathway, MAPK signaling pathway, AMPK signaling pathway, Wnt signaling pathway, and other important KEGG term might be involved in the pathological process of RPL and EMs.

### 3.4 PPI network analysis

**FIGURE 5:**
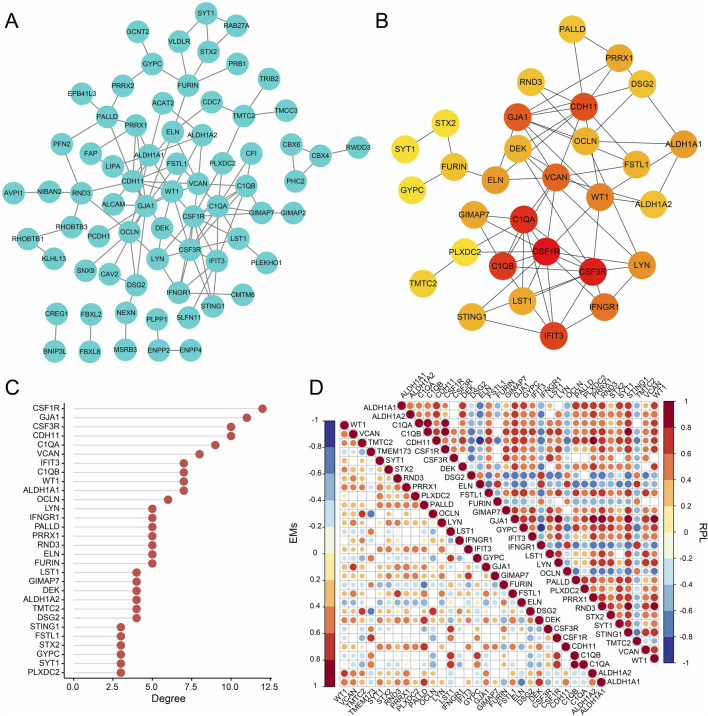
PPI network analysis of the intersection (98 genes) of the key module of RPL and that of EMs (the blue and the brown modules). **(A)** PPI network construction of the 98 genes by the STRING database. **(B)** The subnetwork of the core genes of EMs with comorbid RPL (Top 30 genes by the MCC algorithm). **(C)** The ranking scores of the 30 core genes. **(D)** The expression correlation of the 30 core genes in the combined RPL dataset and the combined EMs dataset.

From the three gene modules related to RPL with EMs, 98 key genes were identified and used to construct a PPI network with the STRING database (Figure 5A). Then, the MCC of each node was calculated and the top 30 genes with the highest MCC were identified as potential hubs which might be closely related to RPL and EMs (Figures 5B, C). Next, we investigated the expression correlation of the 30 core genes in RPL and EMs. The correlation matrices in Figure 5D suggested that most of the 30 genes were positively related in both RPL and EMs.

### 3.5 Identification of the common core genes in RPL and EMs

**FIGURE 6:**
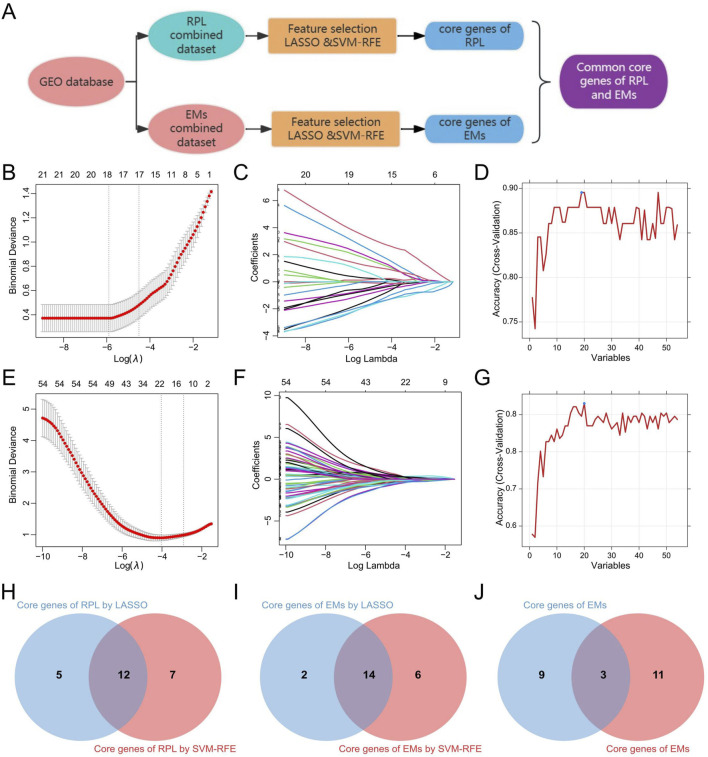
Identification of the common core genes in RPL and EMs. **(A)** Workflow for screening of the common core genes in RPL and EMs. **(B, C)** LASSO regression with ten-fold cross-validation to select the candidate core genes in RPL. **(D)** SVM-RFE result for feature selectin in RPL. **(E, F)** LASSO regression with ten-fold cross-validation to select the candidate core genes in EMs. **(G)** SVM-RFE result for feature selectin in EMs. **(H–J)** The 12 core genes in RPL, the 14 core genes in EMs, and the 3 common core genes in both RPL and EMs selected by LASSO regression and SVM-RFE.

Based on the 30 core genes of the PPI network and the 26 shared DEGS of RPL and EMs, we obtained 54 genes as the core genes for RPL associated with EMs (two duplicate genes were removed), which were then used to perform LASSO and SVM-RFE approaches to get diagnostic biomarkers for disease diagnosis. The workflow for screening design is presented in Figure 6A. Following the 10-fold cross-validation procedure, LASSO regression identified 17 candidate core genes of RPL (Figures 6B, C), and the SVM-RFE method screened 19 candidate core genes in RPL (Figure 6D). After taking the intersection (Figure 6H), a total of 12 core genes were retrieved in RPL. On the other hand, through LASSO regression and SVM-RFE, we obtained 16 and 20 candidate EMs core genes, respectively (Figures 6E–G). The 14 overlapping genes were identified as core genes in EMs (Figure 6I). Finally, we obtained three common core genes in both RPL and EMs by intersecting LASSO regression and SVM-RFE including MAN2A1, PAPSS1, and RIBC2 (Figure 6J).

### 3.6 Diagnostic/therapeutic values in RPL and EMs and the upstream regulations prediction of the three common core genes

**FIGURE 7:**
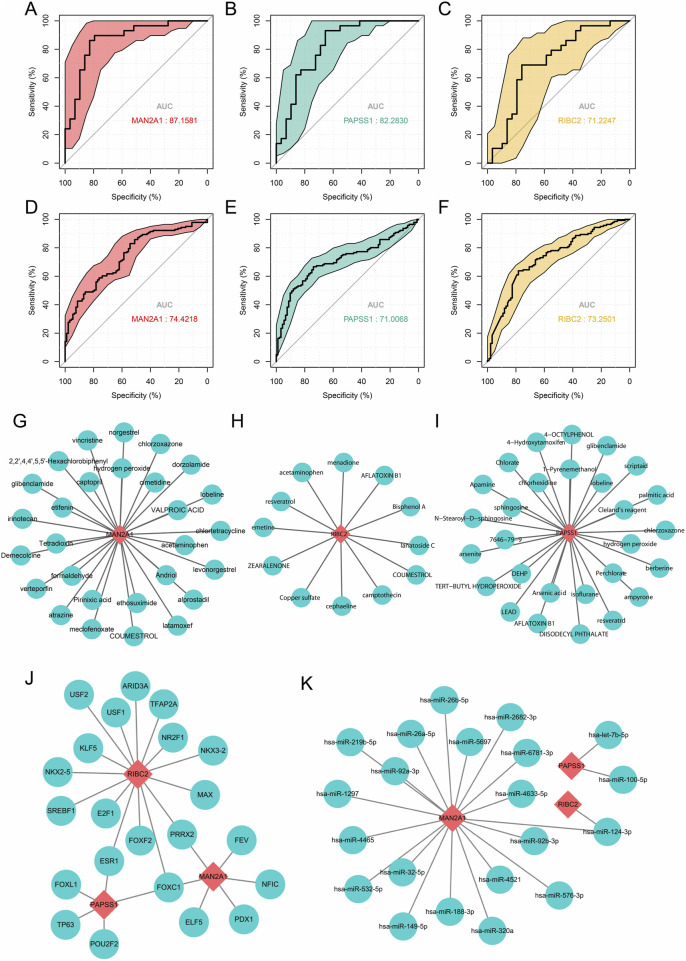
Diagnostic values in RPL and EMs and the upstream regulations prediction of the three common core genes. **(A–F)** ROC curves showing the AUC values with 95%CI of the three diagnostic markers in RPL and EMs. **(G–I)** Potential small drugs that may target MAN2A1, PAPSS1, and RIBC2 predicted by the DSigDB database. **(J, K)** Upstream transcriptors **(J)** and miRNAs **(K)** that may regulate the expression of the three common core genes were predicted by miRNet and miRTarBase.

We drew ROC curves of the three common core genes to evaluate their diagnostic value in RPL and EMs (Figures 7A–F). The area under the curves (AUCs) of individual key genes exceeds 0.7, which denotes all three common core genes had significant diagnostic value in disease discrimination. For therapeutic implications, we inferred the putative small molecules that may perturb the gene expression levels of MAN2A1, PAPSS1, and RIBC2. Consequently, all these three genes were predicted to be targeted by more than 10 therapeutic drugs. A drug-gene interaction network was constructed by Cytoscape for MAN2A1, PAPSS1, and RIBC2, respectively (Figures 7G–I).

Besides, we predicted potential upstream regulations of the three common core genes related to RPL and EMs, which involved transcription factors and regulative miRNA (Supplementary Table S2). Predictive transcription factors of these three core genes were derived from the Jaspar database deposited in the miRNet online application, and putative miRNAs targeting these common core genes were obtained from the miRTarBase database. A transcription factor-core gene network consisting of 22 transcription factors and a miRNA-target network consisting of 21 miRNAs were constructed and visualized by Cytoscape (Figures 7J, K). Conspicuously, RIBC2 and MAN2A1 are the most important nodes in the transcription factor-core network or the miRNA-target network, respectively, indicating their crucial role in both RPL and EMs. These findings could provide new insight into the further exploration of the molecular mechanisms of RPL and EMs.

### 3.7 Evaluation of immune cell infiltration in RPL and EMs

**FIGURE 8:**
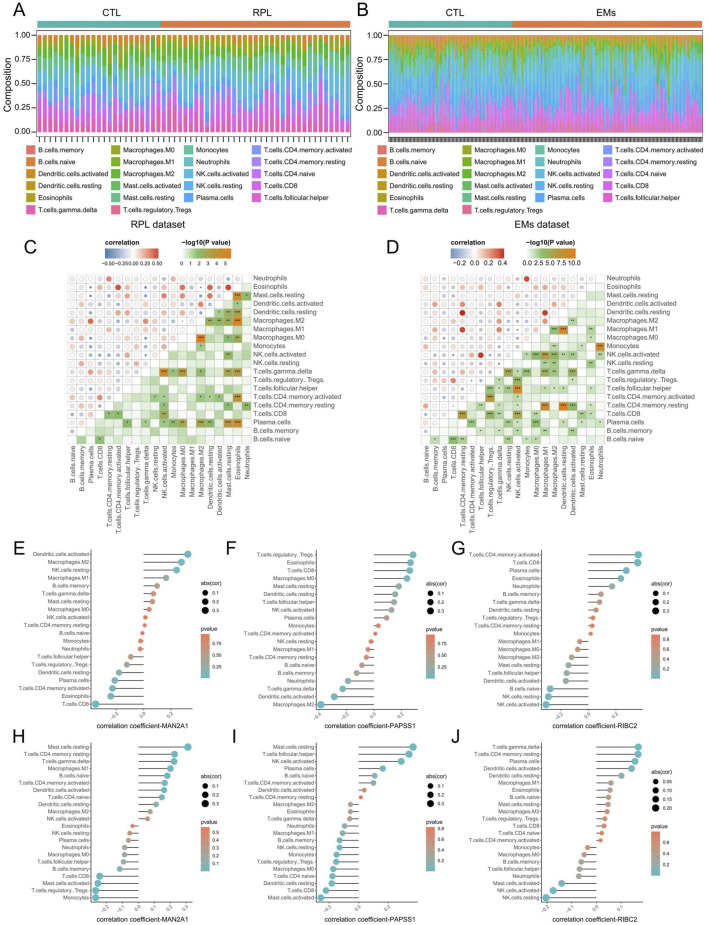
Evaluation of immune cell infiltration in RPL and EMs. **(A, B)** Immune cell landscapes of RPL **(A)** and EMs **(B)** estimated by the CIBERSORTx algorith. **(C, D)** Correlation matrix of immune cell infiltration in RPL **(C)** and EMs **(D)**. Blue indicates negative correlation, and red indicates positive correlation. **(E–G)** Correlation coefficients of MAN2A1, PAPSS1, and RIBC2 with immune cell infiltration in RPL. **(H–J)** Correlation coefficients of MAN2A1, PAPSS1, and RIBC2 with immune cell infiltration in RPL.

Through the CIBERSORTx algorithm, we achieved the landscape of the 22 immune infiltration cells for RPL and EMs (Figures 8A, B). Next, we calculated the correlation of the immune cell infiltration for RPL and EMs. The correlation matrix of immune cell infiltration in RPL and EMs revealed high correlations of the 22 immune infiltration cells in RPL and EMs, which suggested immune infiltration cells may play critical roles in the development of RPL and EMs (Figures 8C, D). The relationship between the three core genes (MAN2A1, PAPSS1, and RIBC2) and immune cells was further investigated using Spearman correlation analysis. As illustrated in Figures 8E–J, distinct results were observed for the correlation analysis of immune cells between the two groups. Specifically, in RPL, MAN2A1 had a positive correlation with Dendritic cells activated (r = 0.32, p = 0.01) and Macrophages M2 (r = 0.28, p = 0.04), while it showed a negative correlation with T cells CD8 (r = −0.35, p = 0.007). PAPSS1 was positively correlated with T cells CD8 (r = 0.26, p = 0.04), T cells Regulatory Tregs (r = 0.28, p = 0.03), Eosinophils (r = 0.26, p = 0.04), and it was negatively correlated with Macrophages M2 (r = −0.38, p = 0.002), Dendritic cells Activated (r = −0.28, p = 0.02). RIBC2 exhibited a positive correlation with Plasma Cells (r = 0.27, p = 0.03), T cells CD8 (r = 0.35, p = 0.005) and T cells CD4 memory active (r = 0.36, p = 0.005), and a negative correlation with NK cell activated (r = −0.30, p = 0.01), B cells Naive (r = −0.27, p = 0.03, NK cells Resting (r = −0.28, p = 0.02) (Figures 8E–G; Supplementary Table S3). On the other hand, for EMs, MAN2A1 and PAPSS1 had a positive correlation with Mast cells resting (r = 0.31, p = 1.36E-06 and r = 0.36, p = 6.55E-09, respectively) and a negative correlation with T cells CD8 (r = −0.24, p = 0.0001). RIBC2 was positively correlated with T cells CD4 memory resting (r = 0.16, p = 0.009), and a negative correlation with NK cell activated (r = −0.17, p = 0.007) (Figures 8H–J; Supplementary Table S4). The above findings indicated that immune cell infiltration had great potential in the occurrence, development, and prognosis of RPL and EMs.

### 3.8 The expression of three hub genes in pan-cancer

Considering the key gene modules of RPL and EMs were enriched in cancer-related KEGG pathways, such as PI3K-Akt signaling pathway, mTOR signaling pathway, p53 signaling pathway, MAPK signaling pathway, cell cycle, and Wnt signaling pathway, etc., which indicate the innate linkage of these diseases and the pathogenesis of cancer, we next analyzed the expression of three core genes in various types of cancer by the TIMER2.0 database (Supplementary Figure S3A–C). Dysregulated expressions were observed for all three core genes in certain cancer types. Notably, MAN2A1 and RIBC2 exhibited significantly differential expression in endometrial cancer compared with adjacent normal tissue, while PAPSS1 had no significant difference. The result suggested that MAN2A1 and RIBC2 may act as a risk or protective factor in the occurrence, development, and prognosis of endometrial cancer.

### 3.9 Comprehensive analysis of RIBC2 and MAN2A1 in endometrial cancer

**FIGURE 9:**
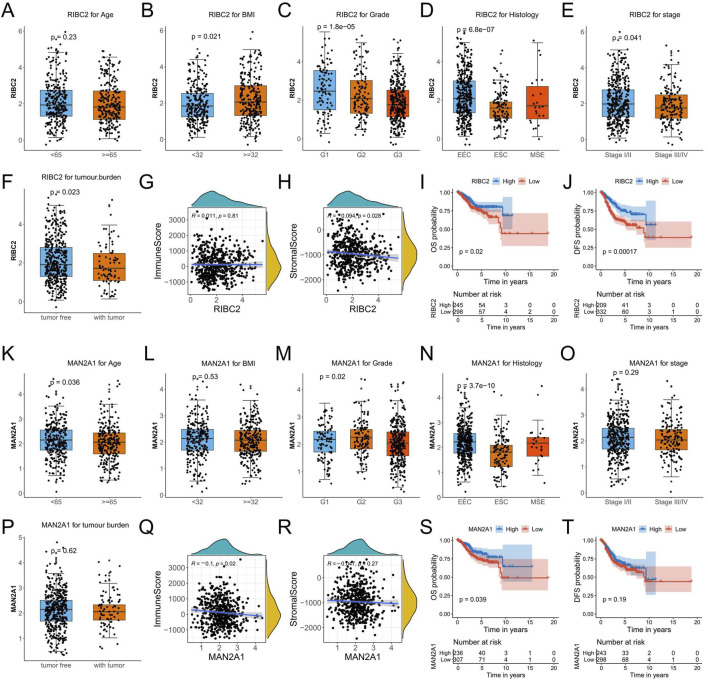
Comprehensive analyses of RIBC2 and MAN2A1 based on the TCGA-UCEC dataset. **(A–F)** Comparison of the RIBC2 expression in subsets of endometrial carcinoma patients with different age, BMI, grade, histology, stage, and tumor burden status. **(G, H)** Spearman correlation analysis between RIBC2 and immune score **(G)** and stromal score **(H)**. **(I, J)** Kaplane-Meier survival plot of OS and DFS for the expression of RIBC2. **(K–P)** Comparison of the MAN2A1 expression in subsets of endometrial carcinoma patients with different age, BMI, grade, histology, stage, and tumor burden status. **(Q, R)** Spearman correlation analysis between MAN2A1 and immune score **(Q)** and stromal score (R). **(S, T)** Kaplane-Meier survival plot of OS and DFS for the expression of MAN2A1.

To investigate the relevance to clinical variables, the RIBC2 expression in the RNA-seq profile of endometrial carcinoma patients was assessed by different age, BMI, grade, histology, stage, and tumor burden status. Consequently, except for age, the RIBC2 expression was significantly associated with BMI, grade, histology, stage, and tumor burden status (Figures 9A–F). Meanwhile, the MAN2A1 expression was significantly associated with age, grade, and histology. There were no significant associations in BMI, stage, and tumor burden status (Figures 9K–P). Moreover, while RIBC2 exhibited a negative correlation with stromal score (Figure 9H), MAN2A1 was negatively associated with immune score (Figure 9Q). The results suggested their predominant roles in the initiation of carcinogenesis and the involvement of the heterogeneous tumor microenvironment in EC. For Kaplan-Meier survival analyses, both the OS survival and the DFS survival were shown to be significantly higher in the high-expression group of RIBC2 (Figures 9I, J). Similarly, the high-expression of MAN2A1 group showed a significantly superior OS survival rate than that of the low-expression group of MAN2A1 (Figures 9S, T).

### 3.10 Protein expression of RIBC2, MAN2A1, and PAPSS1 in the clinical samples of EMs

To further verify the expression levels of the abovementioned core genes in clinical samples, we enrolled three ectopic endometriotic lesion samples and three endometrial samples for immunohistochemistry assays. As the result, protein expression analysis of endometrial tissue in the hyperplasia stage and EMs showed that RIBC2 gene was expressed in both glandular cells and stromal cells, MAN2A1 was mainly expressed in the cytoplasm of glandular cells, and PAPSS1 was mainly expressed in the nucleus of glandular cells. All three proteins were abnormally expressed compared to endometrial tissue at the hyperplasia stage (Supplementary Figure S4).

## 4 Discussions

Endometriosis is a chronic inflammatory disease, mainly characterized by the colonization and growth of endometrial cells outside the uterine cavity. This process seriously endangers women’s physical and mental health. Despite the existence of several proposed theories, including retrograde menstruation, endometrial stem cell implantation, residual Mullerian abnormalities, and body cavity metaplasia, there is currently no established theory that can fully explain the pathogenesis of all types of endometriosis lesions. Additionally, the definitive diagnosis and treatment of endometriosis often entails a traumatic experience due to the necessity for surgical procedures to visualize the abdominal or pelvic cavity and to remove the lesions. Endometriosis alters the internal milieu of the uterus and pelvis, thereby increasing the risk of early miscarriage and the development of RPL.

Currently, bioinformatics employs a multidisciplinary approach, drawing upon biology, computer science, information engineering, and other fields, to analyze and integrate copious amounts of data, with applications in life science research. In this study, we employed the WGCNA methodology to identify putative biomarkers and therapeutic targets employing clustering highly correlated genes and analyzing the correlation between these clusters and clinical features. The gene modules related to EMs and RPL were retrieved using WGCNA, resulting in the identification of 98 shared genes. The PPI network for these genes was constructed using the STRING database, and the key genes were subsequently identified through LASSO and SVM-RFE. The intersection of these three methods yielded three common core genes in RPL and EMS, MAN2A1, PAPSS1, and RIBC2.

3′-Adenosine 5′-phosphate sulfate (PAPS) synthetase 1 (PAPSS1) catalyzes the synthesis of PAPS from ATP and inorganic sulfate (SO₄^2^⁻) through the reaction of ATP thiolase and adenosine 5-phosphate sulfate kinase, which serves as the substrate for cellular sulfonation reactions. PAPSS is expressed widely throughout the human body and may be involved in the sulfation process of various organs (Xu et al., 2000). Sulfuration regulates numerous biological effects through the modification of proteins, carbohydrates, and lipids. An illustrative example is its role in mediating the initial interaction between leukocytes and endothelial cells (Pouyani and Seed, 1995; Wilkins et al., 1995). This process may be related to early ectopic growth of endometrium. Sulfation represents a major pathway of estrogen metabolism (Foster, 2021). Estradiol sulfate, which can be reactivated by the desulfurization of estrogen sulfatase. The overexpression of PAPSS1 has been demonstrated to impede estrogen-stimulated cell proliferation (Xu et al., 2012). The sulfation mediated by PAPSS1 is implicated in the process of DNA damage repair (Leung et al., 2015), which, in turn, affects the expression of estrogen-responsive genes (Sun L. et al., 2023). The close relationship between papss1 and hormones also suggests that PAPSS1 plays an important role in EMs and RPL, which are closely involved in hormone regulation. Polymorphisms within the ATP sulfurylase domain of the PAPSS1 gene are also strongly correlated with both liver cancer susceptibility and the efficacy of drug therapy (Shih et al., 2009). It would be of interest to ascertain whether this structural polymorphism exerts disparate effects on estrogen metabolism.

Mannosidase alpha class 2A member 1 (MAN2A1), also referred to as MAN2A1, is a glycosidase found within the Golgi apparatus. Its primary function is to cleave N-glycan precursors into mannose (Man) residues, rendering it an essential substrate for the formation of complex N-glycans (Moremen and Robbins, 1991; Misago et al., 1995). Glycan biosynthesis is essential for the maintenance of cellular structure and function and also plays a pivotal role in cell signaling, immune responses, and inflammatory processes. Concurrently, MAN2A1 is a crucial enzyme in the glycosylation of mature membrane proteins, which is essential for ensuring signal transmission and material transport (Fagerberg et al., 2014; Shi et al., 2020). Abnormal MAN2A1 expression has also been observed to affect pathways related to hedgehog, epidermal growth factor, and transforming growth factor signaling (Anvarian et al., 2019). N-glycans are involved in the expression and function of immune cell surface glycoproteins and affect the binding of immune receptors and ligands (Lyons et al., 2015; Li et al., 2018).

The RIB43A domain with coiled-coils 2 (RIBC2) is also known as TRIB2. The TRIB protein family belongs to a highly conserved type of serine/threonine pseudokinase. In the absence of serine/threonine protein kinase activity, the domain is involved in cell signal transduction, acting as a scaffold protein to regulate protein stability and some protein kinase activities. This affects a variety of cellular processes, including proliferation, differentiation, cell cycling, and cell death (Boudeau et al., 2006; Hill et al., 2015; Monga et al., 2022). The TRIB gene family comprises three homologous genes, TRIB1, TRIB2, and TRIB3. Among them, TRIB2 is mainly located in the cytoplasm and participates in the pathogenesis and progression of various tumors (Monga et al., 2022; Hill et al., 2015). TRIB2 mainly regulates target genes at the post-transcriptional level, such as the modification of ubiquitinated proteins and the subsequent alteration of their functionality, as well as the modulation of target genes through the classical AKT or MAPK pathways (Eyers et al., 2017).

Notably, all three of these core markers (MAN2A1, PAPSS1, and RIBC2) in RPL and EMs showed high potential for early detection of both diseases with AUCs greater than 0.7. Thus, more future studies are expected to develop non-invasive biomarkers for the diagnosis of RPL and EMs based on the three core genes, including cell-free and exosomal-derived miRNAs, circRNAs or DNA/RNA methylation markers. Moreover, we evaluated the utility of MAN2A1, PAPSS1, and RIBC2 for serving as molecular targets in predicting novel drugs with DSigDB database. Remarkably, all of them were predicted to be targeted by more than 10 small compounds, suggesting the great potential in clinical drug discovery. Taken together, the identified core genes are promising targets with significant implications for both the diagnosis and treatment of RPL and EMs in clinical practice.

The correlation analysis of immune cells exhibited that the infiltration of immune cells was closely related to the development of RPL and EMs. The analysis of MAN2A1, PAPSS1, and RIBC2 genes and immune cell infiltration found that the three genes were also closely associated with immune cell infiltration in the two diseases. MAN2A1 has been previously identified as a regulator of the inflammatory response and autoimmune diseases. For instance, MAN2A1 is involved in the development and progression of glomerulonephritis (Chui et al., 2001). Mice lacking MAN2A1 exhibit a deficiency in N-glycans on red blood cells, resulting in anemia and the development of a late-onset autoimmune disease that closely resembles systemic lupus erythematosus (SLE) (Chui et al., 1997; Chui et al., 2001). At the same time, MAN2A1 may affect vascular development and vascular deformation through NAC (antioxidant like N-acetyl cysteine NAC) (Herrera et al., 2017; Ningappa et al., 2021). It was postulated that the effects of MAN2A1 on endothelial cell growth and vascular deformation may be closely related to ectopic endometrial growth and RPL. Additionally, TRB2 has been demonstrated to exhibit high expression in T cells within normal hematopoietic cells, particularly in lymphoid tissues. Its elevated expression is linked to the T cell receptor (TCR) signaling pathway, particularly the Notch signaling pathway (Breit et al., 2006; Hannon et al., 2012). TRIB2 has been shown to activate p38, thereby reducing the chemotherapy resistance and disease progression of myeloid leukemia (Salome et al., 2018). Collectively, existing evidence suggests that these three genes play a role in regulating immune cells.

Abnormal immune function has been linked to the occurrence and development of tumors. The infiltration of immune cells has also been implicated in regulating the development of tumors. Given that these three genes are engaged in the regulation of immune cells, as well as the significant enriched KEGG pathways of well-known cancer-related terms that appeared in EMs and RPL, such as PI3K-Akt signaling pathway, p53 signaling pathway, MAPK signaling pathway, cell cycle and Wnt signaling pathway, we undertook a pan-cancer analysis of their expression to ascertain whether endometriosis is associated with endometrial cancer. Our findings elucidated that RIBC2 and MAN2A1 are aberrantly expressed in EC. The TCGA-UCEC dataset analysis revealed a correlation between RIBC2 expression and various clinicopathological variables, including body mass index (BMI), grade, histology, stage, and tumor burden status. The Spearman correlation analysis uncovered a negative correlation between RIBC2 and stromal score. The Kaplan-Meier survival plot of OS and DFS for the expression of RIBC2 was found to be associated with both OS and DFS, which is consistent with the published literature. Studies suggest that TRIB2 overexpression negatively regulates the activity of the WNT signaling pathway and inhibits WNT-mediated transcriptional activity in hepatocellular carcinoma (HCC), which is closely related to patient prognosis (Wang et al., 2013). In HCC, TRIB2 has proven to inhibit Wnt signaling and tumorigenesis by regulating the stability of βTrCP, COP1, and Smurf1 (Xu et al., 2014). In colorectal cancer, TRIB2 is observed to block cell senescence through ap4/p21 signaling (Hou et al., 2018).

Similar outcomes were observed with regard to MAN2A1 expression, which was found to be significantly associated with age, grade, and histology in EC. It has been demonstrated that MAN2A1 is markedly decreased in colorectal cancerous tissues in comparison to adjacent normal tissues. Furthermore, it has been observed that MAN2A1 is downregulated in metastatic colorectal cancer in contrast to non-metastatic colorectal cancer (CRC). A significant negative correlation was observed between increased MAN2A1 expression and the progression of CRC (Wang et al., 2022). The fusion of MAN2A1 with the tyrosine kinase FER results in the transformation of MAN2A1 into an oncogene, leading to the development of multiple cancers. MAN2A1-FER fusions have been identified in prostate cancer, liver cancer, esophageal cancer, and other types of malignant tumors (Yu et al., 2014; Chen et al., 2017; Yu et al., 2021). The MAN2A1-FER fusion facilitates the transport of the FER kinase to the Golgi apparatus, where it activates and promotes cancer through the epidermal growth factor receptor (EGFR) signaling pathway (Lee et al., 2014).

In conclusion, we employed multiple bioinformatics approaches to identify three core genes, MAN2A1, PAPSS1, and RIBC2, which were demonstrated to play a pivotal in the occurrence and development of EMs and RPL. Subsequent analysis confirmed all these genes exhibited excellent performance in the diagnosis of EMs and RPL, which were also found to be involved in the regulation of immune cell infiltration. Interestingly, two core genes, RIBC2 and MAN2A1, were further identified as key players in the onset and progression of cervical cancer. These findings may prove beneficial in providing potential targets for the diagnosis and prognosis of EMs and RPL patients, as well as offering insights into novel treatment strategies.

## Data Availability

The datasets presented in this study can be found in online repositories. The names of the repository/repositories and accession number(s) can be found in the article/Supplementary Material.
