# Supplementary material for: Potential biomarkers and immune infiltration linking endometriosis with recurrent pregnancy loss based on bioinformatics and machine learning
Source: Front Mol Biosci. 2025 Feb 3;12:1529507. doi: 10.3389/fmolb.2025.1529507 (PMC11830612; doi:10.3389/fmolb.2025.1529507)
Supplement: Supplementary file 5 [file Image1.pdf]

Supplementary Material

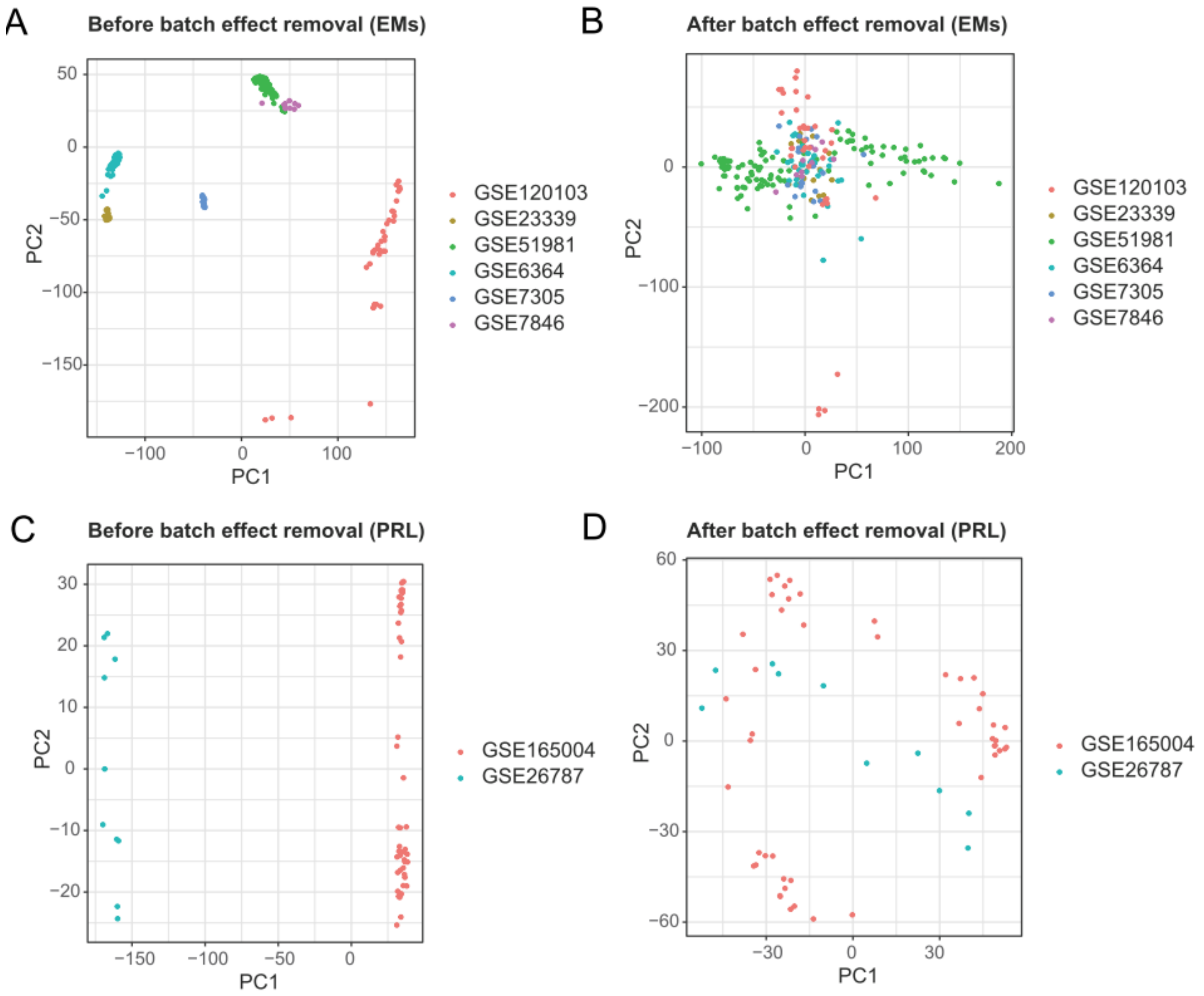

Supplementary Figure 1 Principal component analysis (PCA) for the expression files of EMs (A, B) and RPL (C, D) of multiple array datasets before and after batch effect removal.

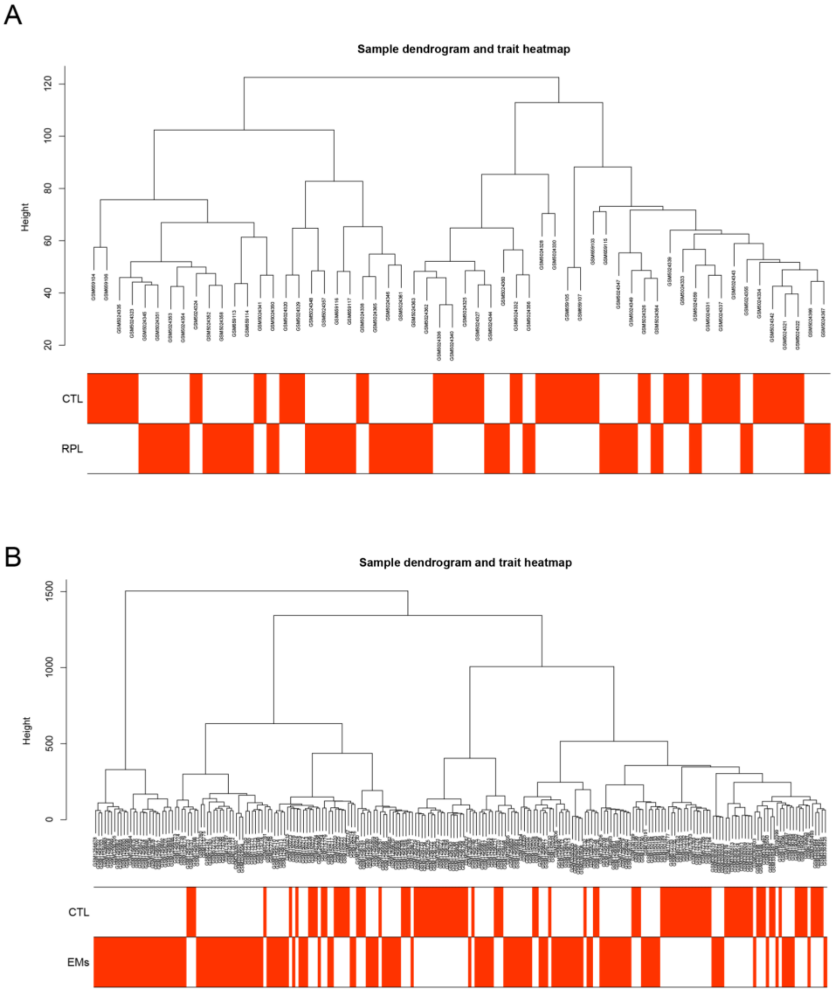

Supplementary Figure 2 Sample clustering trees of RPL (A) and EMs (B). RPL, recurrent pregnancy loss; EMs, Endometriosis.

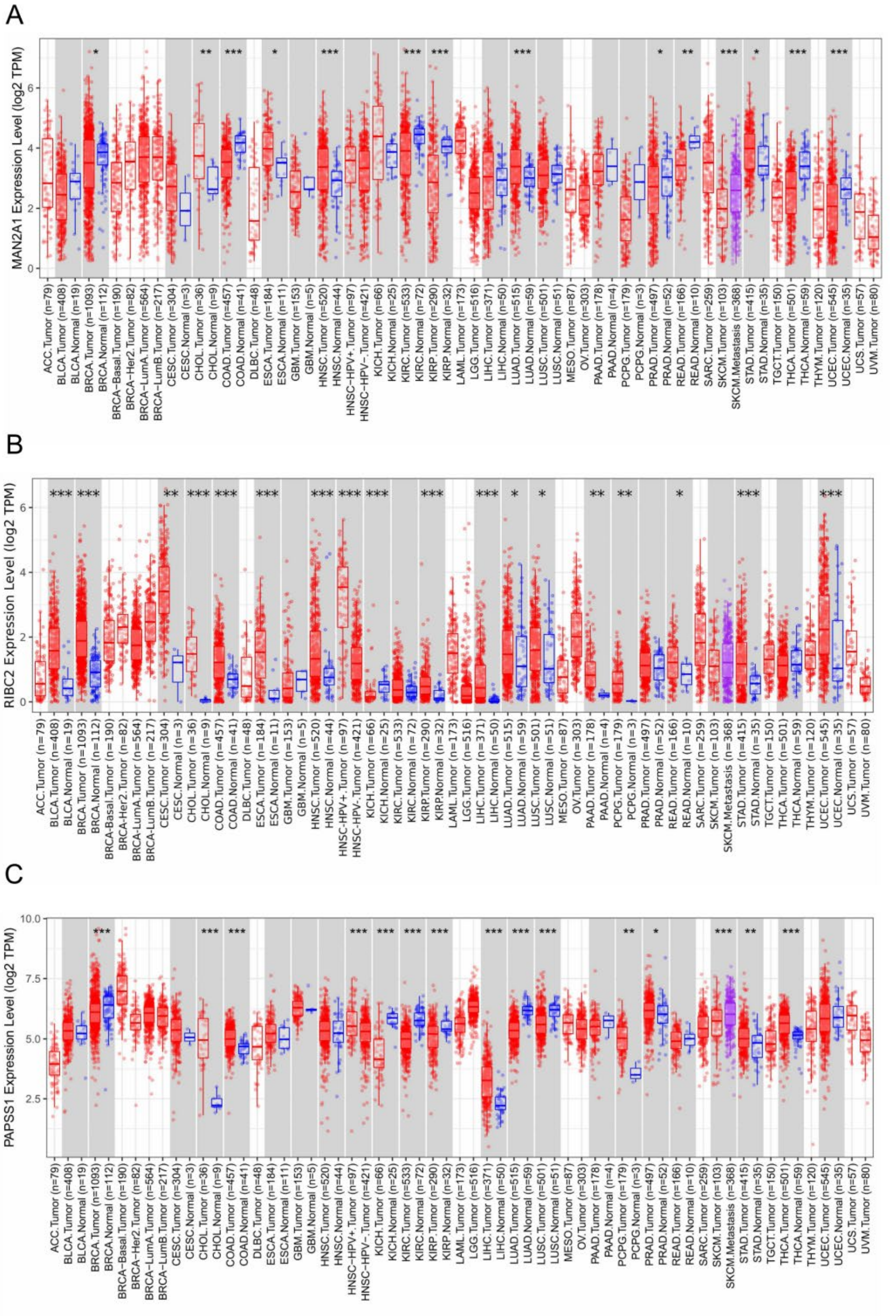

Supplementary Figure 3 The expression of MAN2A1, RIBC2, and PAPSS1 in various types of cancers by the TIMER2.0 database.

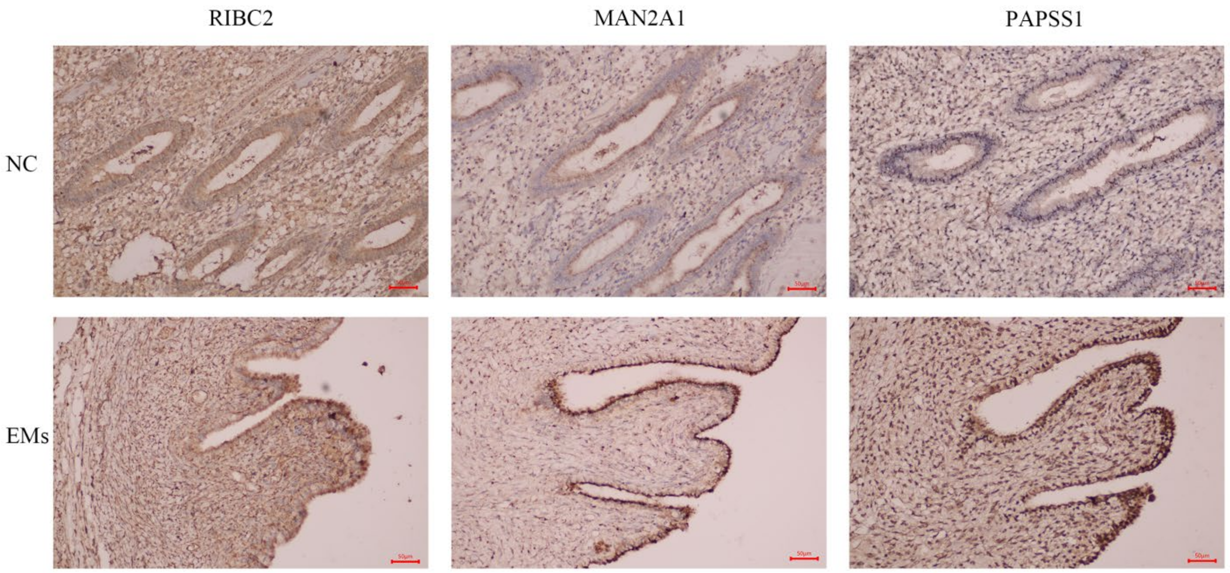

Supplementary Figure 4 Representative images of immunohistochemistry (IHC) showing the abnormally expression of RIBC2, MAN2A1, and PAPSS1 in EMs at protein level.
